# Retinoid-Binding Proteins: Similar Protein Architectures Bind Similar Ligands via Completely Different Ways

**DOI:** 10.1371/journal.pone.0036772

**Published:** 2012-05-04

**Authors:** Yu-Ru Zhang, Yu-Qi Zhao, Jing-Fei Huang

**Affiliations:** 1 State Key Laboratory of Genetic Resources and Evolution, Kunming Institute of Zoology, Chinese Academy of Sciences, Kunming, Yunnan, China; 2 Graduate School of Chinese Academy of Sciences, Beijing, China; 3 Kunming Institute of Zoology-Chinese University of Hongkong Joint Research Center for Bio-Resources and Human Disease Mechanisms, Kunming, China; University of South Florida College of Medicine, United States of America

## Abstract

**Background:**

Retinoids are a class of compounds that are chemically related to vitamin A, which is an essential nutrient that plays a key role in vision, cell growth and differentiation. In vivo, retinoids must bind with specific proteins to perform their necessary functions. Plasma retinol-binding protein (RBP) and epididymal retinoic acid binding protein (ERABP) carry retinoids in bodily fluids, while cellular retinol-binding proteins (CRBPs) and cellular retinoic acid-binding proteins (CRABPs) carry retinoids within cells. Interestingly, although all of these transport proteins possess similar structures, the modes of binding for the different retinoid ligands with their carrier proteins are different.

**Methodology/Principal Findings:**

In this work, we analyzed the various retinoid transport mechanisms using structure and sequence comparisons, binding site analyses and molecular dynamics simulations. Our results show that in the same family of proteins and subcellular location, the orientation of a retinoid molecule within a binding protein is same, whereas when different families of proteins are considered, the orientation of the bound retinoid is completely different. In addition, none of the amino acid residues involved in ligand binding is conserved between the transport proteins. However, for each specific binding protein, the amino acids involved in the ligand binding are conserved. The results of this study allow us to propose a possible transport model for retinoids.

**Conclusions/Significance:**

Our results reveal the differences in the binding modes between the different retinoid-binding proteins.

## Introduction

Vitamin A is an essential nutrient that plays a key role in vision, cell growth and differentiation, and embryonic development. Vitamin A is ingested from dietary sources as a retinyl ester or synthesized from β-carotene and is stored in the liver as a retinyl ester until it is mobilized for delivery to various target tissues. Retinol is one of the forms of vitamin A obtained from foods of animal origin. Retinal (retinaldehyde), the aldehyde derived from retinol, is essential for vision, while retinoic acid is essential for skin health and bone growth. These chemical compounds are collectively known as retinoids and possess the same structural motif (i.e., all-trans double bonds) found in retinol. Structurally, all retinoids possess a β-ionone ring and a polyunsaturated side chain containing an alcohol, an aldehyde, a carboxylic acid group or an ester group [1]. Because of their chemical instability and fairly low solubility in aqueous media, retinoids must be bound by specific proteins in bodily fluids and within cells. Plasma retinol-binding protein (RBP) and epididymal retinoic acid binding protein (ERABP) carry retinoids in bodily fluids, while cellular retinol-binding proteins (CRBPs) and cellular retinoic acid-binding proteins (CRABPs) carry retinoids within cells [2].

RBP, ERABP, CRBPs (CRBP I, II, III, and IV) and CRABPs (CRABP I and CRABP II) belong to the lipocalins superfamily in the Structural Classification of Proteins (SCOP) database [3]. Although they differ both in sequence and function, all members of the lipocalins superfamily contain a six- or eight-stranded β-barrel as part of their tertiary structure and a highly conservative motif, the short conserved region (SCR), as part of their amino acid sequence [4]. In the SCOP, RBP and ERABP belong to the retinol-binding protein-like (RBP) family. CRBPs and CRABPs belong to the fatty acid-binding protein-like (FABP) family. RBP is the specific carrier for retinol (vitamin A alcohol) in the blood. It delivers retinol from the liver stores to peripheral tissues. In plasma, the RBP-retinol complex interacts with transthyretin, which prevents it from being filtered out of the blood by the kidney glomeruli. The basic structural framework of RBP consists of an eight-stranded up-and-down β-barrel onto which a carboxy-terminal α-helix is attached [5], [6]. ERABP in the lumen of the epididymis is required for sperm maturation and binds both all-trans retinoic acid and 9-cis retinoic acid. Like all other lipocalins, ERBP contains an eight-stranded up-and-down β-sheet core, which twists into a barrel. One end of the barrel is closed off by amino acid side chains in the barrel interior and amino acid side chains from the amino terminus portion of the protein, which wrap across the back side of the barrel [7]. As with other proteins in the FABP family, CRBPs and CRABPs have an overall tertiary structure comprised of 10 anti-parallel β-strands, which are themselves formed from two five-stranded β-sheets arranged approximately perpendicular to each other [8]. CRBP I, II, III and IV are highly homologous proteins, but have distinct tissue distributions and retinoid-binding properties. Among these CRBPs, mammalian CRBP I and II are the best-characterized members of the CRBP family and are known to bind all-trans retinol and all-trans retinal with a high affinity but not all-trans retinoic acid [2], [7], [9], [10], [11]. Cellular retinoic acid-binding proteins may regulate the interactions between retinoic acids and their nuclear receptors by regulating the concentration of retinoic acids present [12], [13].

In general, if two ligands are structurally similar, the orientation and mode of binding for these ligands in related proteins is typically conserved. That is, a majority of the ligand pairs occupy the same space in the binding sites [14]. Interestingly, although RBP, ERABP, CRBPs and CRABPs have homologous structural motifs and overlapping ligand specificity, they have different binding mechanisms. This demonstrates an important principle, namely, that similar protein architectures can be used to bind identical ligands via completely different ways. For example, Kleywegt was the first to discover that ERABP and CRABP bind retinoic acid with different orientations. However, they gave no explanation regarding the evolutionary of the two binding modes [13]. Although many researchers are interested in the different mechanisms of retinoid transport [7], [13], [15], most studies focus on comparisons within one family of proteins, such as comparisons of RBP with ERABP and CRABP with CRBP. To date, there are no exhaustive studies on the mechanisms of retinoid transport that rely on structural analysis.

The aim of this study is to investigate the differences in the various retinoid transport mechanisms. First, we compare the structures of the transport proteins and the differences in their sequences. Then, we analyze the conservation of the retinoid binding residues in the transport proteins. Finally, we propose a possible retinoid transport model and support the model with evidence from molecular dynamics simulations. This study may be useful in delineating the transport mechanism of retinoids.

## Results and Discussion

### Retinoid transport proteins bind identical ligands via different ways

**Figure 1:**
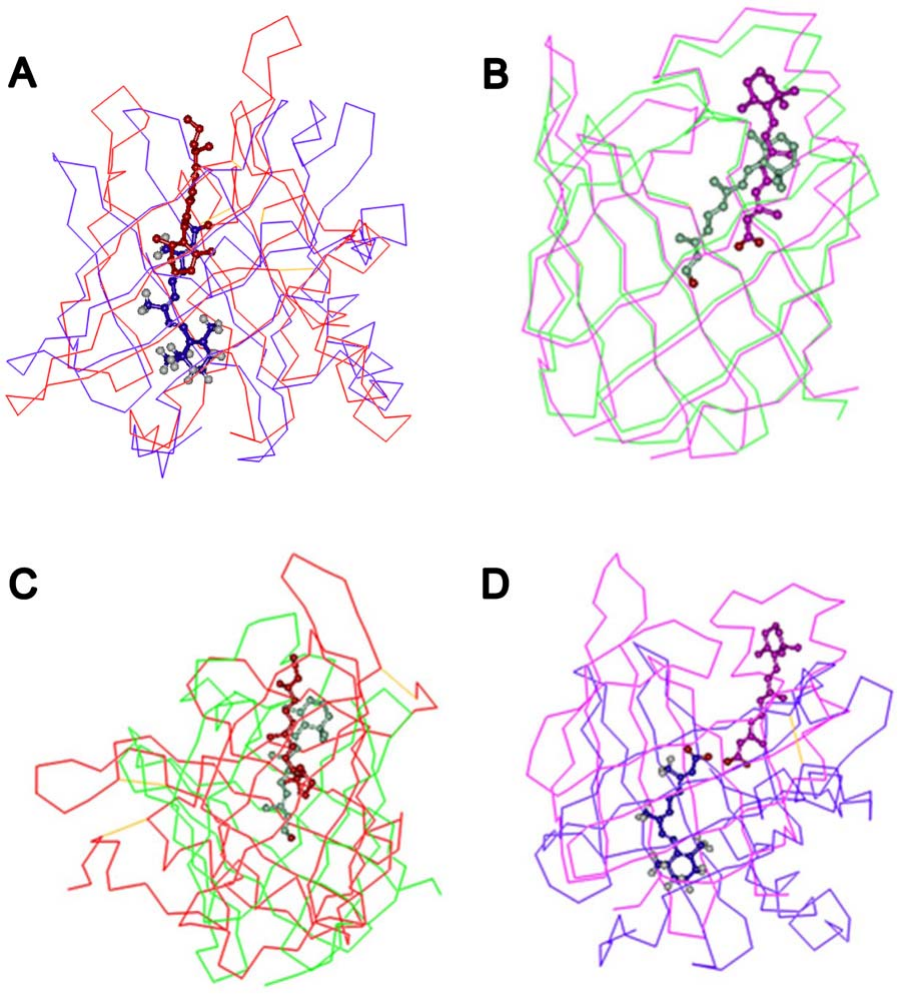
Comparison of retinoid binding in their transport proteins. In the same protein family and subcellular location, retinol and retinoic acid have the same binding orientation (A, B), while in different protein families and subcellular locations, the binding orientations of the ligands are completely different (C, D). (a) In both of the extracellular proteins (RBP, ERABP), the β-ionone ring of the ligand is positioned in the center of the barrel with the isoprene tail extending along the barrel axis pointing toward the solvent. (b) The orientation of the ligand is, therefore, opposite to that in the corresponding intracellular retinoid-binding proteins (CRBPs and CRABPs). The red, blue, green and pink lines indicate RBP (PDB code: 1brp), ERABP (PDB code: 1epb), CRBP (PDB code: 1crb), and CRABP (PDB code: 1cbs) protein structures, respectively. The colors representing retinol and retinoic acid correspond to different transport protein colors.

As their names imply, RBP, ERABP, CRBPs and CRABPs bind similar hydrophobic ligands, including retinol, retinal and retinoic acid, in their interior. Structurally, these proteins belong to the lipocalins superfamily according to the SCOP, which have either a closed or open barrel structural framework consisting of eight to ten anti-parallel β-strands. Meanwhile, all retinoids have both a β-ionone ring and a polyunsaturated side chain containing an alcohol, an aldehyde, and a carboxylic acid group or an ester group. Although RBP, ERABP, CRBPs and CRABPs bind similar ligands (RBP and CRBPs both bind retinol; ERABP and CRABPs both bind retinoic acid), the protein-ligand binding patterns are very different among them (Fig. 1). Proteins of the same family and subcellular location have the same binding orientations. However, when comparing different families of proteins and subcellular locations, the binding orientations of retinoids are completely different. In both of the extracellular retinoid-binding proteins (RBP and ERABP), the β-ionone ring of the ligand is positioned in the center of the barrel with the isoprene tail extending along the barrel axis pointing toward the solvent. However, the orientation of the ligand is opposite to that of the corresponding intracellular proteins (CRBPs and CRABPs).

### The difference of retinoid binding mechanisms among transport proteins within the same family

In the RBP family, RBP and ERABP share a low sequence similarity (21.9%), and display various structural differences (Fig. 1A). In addition to the up-and-down β-barrel, RBP has only one C-terminal α-helix, but ERABP has two. These differences are presumably necessary to allow RBP to be specific for retinol, but not retinoic acid. Structural comparison shows that none of the amino acid residues that form the ligand-binding cavity in RBP is conserved in ERABP. In RBP, retinol binds to a region that is close to the surface of the protein. However, in ERABP, the binding site is deeper in the barrel. The ligand specificity of ERABP is greater than that of RBP. At the portion of the ligand binding site that interacts with the polar tail of the ligand, electrostatic interactions determine the binding specificity [7]. Our dynamics simulations showed that the binding affinity (Table S1) between ERABP and retinoic acid (binding energy: −105.47 kcal/mol) is stronger than that between RBP and retinol (binding energy: −86.83 kcal/mol) (p = 1.83×10^−4^).

In the FABP family, the sequence similarity between CRBP and CRABP is 41% (Table S2). A comparison of their structures shows that their β-sheets are highly superposed (Rmsd = 1.5 Å) (Table S3). However, in CRABP, the retinoic acid binds near the entrance of the barrel, which is higher than for retinol (Fig. 1B). It has been suggested that a trio of residues determine the binding specificity of CRBPs and CRABPs for their ligands [16], [17]. In most FABPs and all CRABPs, three residues (Argl06, Arg126 and Tyr128) that interact with the carboxylate of the bound fatty-acid ligand are highly conserved. In CRBPs, the structurally equivalent residues were altered to be Gln108, Gln128 and Phel30 [18]. These mutations may be necessary to allow RBP to be specific for retinol but not retinoic acid. Thus the retinoid binding site differs significantly between the two types of proteins. In addition, none of the ligand-binding amino acids is conserved between CRBPs and CRABPs (Fig. 1B).

Deciphering the sequence code for protein folding requires the ability to determine which residues are essential for specifying a given fold. Many residues in a protein confer functional capacities, but others may mediate properties cruciality for the success of a protein in its cellular environment (e.g., solubility, biosynthesis, turn-over) [8]. We conclude that the striking structural homology across many organisms, cell types, and ligand shapes is preserved because of the common role of these proteins—to bind relatively large retinoids that present highly hydrophobic surfaces.

### The difference modes of retinol binding for RBP and CRBPs

Despite binding the same ligand, retinol, the sequences of RBP and CRBPs have a low sequence similarity (9.34%) (Table S2). Moreover, structural alignment shows that significant structural differences are apparent (Rmsd = 4.4 Å) (Fig. 1C and Table S3). There is no overlap in the secondary structures of RBP and CRBPs. In addition to an inverted binding orientation of retinol, the polyunsaturated side chain of retinol in cellular retinol-binding proteins is deeper in the cavity (Fig. 1C).

**Figure 2:**
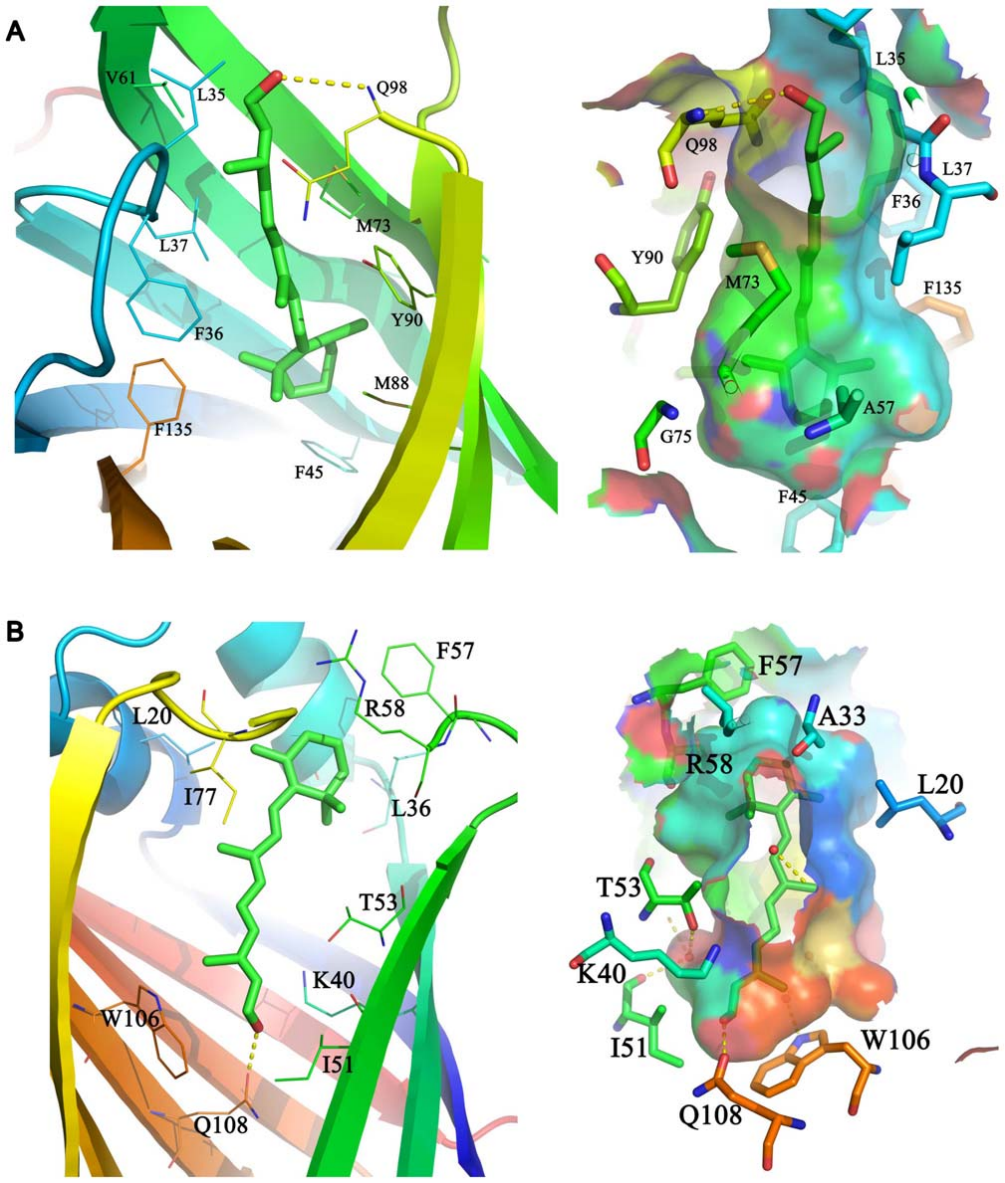
The retinol-binding cavity in RBP and CRBP. Ligand binding sites show as cartoon and transparent surface respectively. The binding site surface is illustrated by meshed colors according to the electrostatic potential. We show the binding cavity in different directions to facilitate the observation of the polar interaction between the retinol and the transport proteins. (A) The yellow dashed line is the polar interaction between the retinol –OH and GLN98 in RBP. (B) The yellow dashed line is the polar interaction between the retinol –OH and GLN108 in CRBP. Water molecules (red balls) adjacent to the retinol –OH form polar interactions between water molecules and other water molecules or side chain amino acids (left picture).

The contacts between the cyclohexene ring and the polyene side chain of retinol and the amino acid residues lining the barrel of plasma RBP are shown in Figure 2A. All of these amino acids are highly conserved in RBP apart from Gln98, which changes to Glu in Xenopus laevis. The interactions between RBP and retinol are mainly hydrophobic, and except for the methyl groups of the polyene side chain, the C19 and C20 atoms are relatively close to polar groups. The hydroxyl group of the retinol side chain is located at the entrance of the RBP barrel and its oxygen atom participates in polar interactions with Q98 and water. The structural comparison between the liganded and unliganded forms of RBP did not reveal significant conformational changes. The most significant difference between the two forms is a conformational change involving residues 34 to 37 [6].

In cellular retinol-binding proteins, the all-trans retinol has a planar conformation in which the hydrogen atom of the hydroxyl group bonds to the side chain of glutamine 108. This interaction explains preference of CRBP for binding retinol rather than retinal. The β-ionone ring at the entrance of the CRBP barrel is surrounded by an amino acid side chain (Fig. 1C). For the most part, the binding cavity conforms to the van der Waal's surface of the retinol. The only polar group found in retinol is the hydroxyl end, which bonds with the side chain of Glu108 (Fig. 2B).

Four types of cellular retinol-binding protein (CRBP I, II, III, and IV) with distinct tissue distributions and retinoid binding properties have been structurally characterized so far [9], [11]. Structural superimposition of these proteins demonstrated that the retinol binding pocket residues are highly superimposable. Indeed, the relative positions of the binding residues in the four types of proteins are nearly same. Moreover, the residues lining the retinol binding site are either identical or chemically conserved in CRBP I, II, III and CRBP IV. The only exception is Q108 whose amide group hydrogen bonds with the hydroxyl group of retinol in CRBP I and most known CRBP II proteins. In chicken and xenopus laevis CRBP II, amino acid 108 is mutated to histidine. The Q108 residue is replaced by histidine in CRBP III and CRBP IV (Fig. S1). This result is consistent with Folli's study on similarities of ligand binding between CRBP I, II, and III [2]. The Q-H switch at position 108 may have functional significance. In fact, H108 protonation might occur after the protein is exposed to a weakly acidic microenvironment or as the consequence of protein conformational change.

### The difference in the retinoic acid binding modes of ERABP and CRABPs

Although both proteins bind retinoic acid, the sequence and structure of ERABP and CRABPs are significantly different (e.g., sequence identity = 15.85%, Rmsd = 3.2 Å) (Table S2 and S3). Moreover, the binding sites for retinoic acid are at very different positions (Fig. 1D). As the structure alignment shows, the retinoic acid –COOH group is in an opposite orientation in the ERABP and CRABP barrels. Although they both have polar sites for binding the –COOH group, the binding sites have no conserved amino acids. Furthermore, the conformation of retinoic acid is changed in different binding proteins. In ERABP, the ligand is clearly sickle shaped, and all-trans retinoic acid adopts an 8-cis structure, where the C7–C8–C9–C10 torsion angle is equal to 0° in the binding cavity [7]. However, in CRABP, the all-trans-retinoic acid is nearly flat with the ionone ring showing a significant deviation (−33°) from the cis conformation [13].

**Figure 3:**
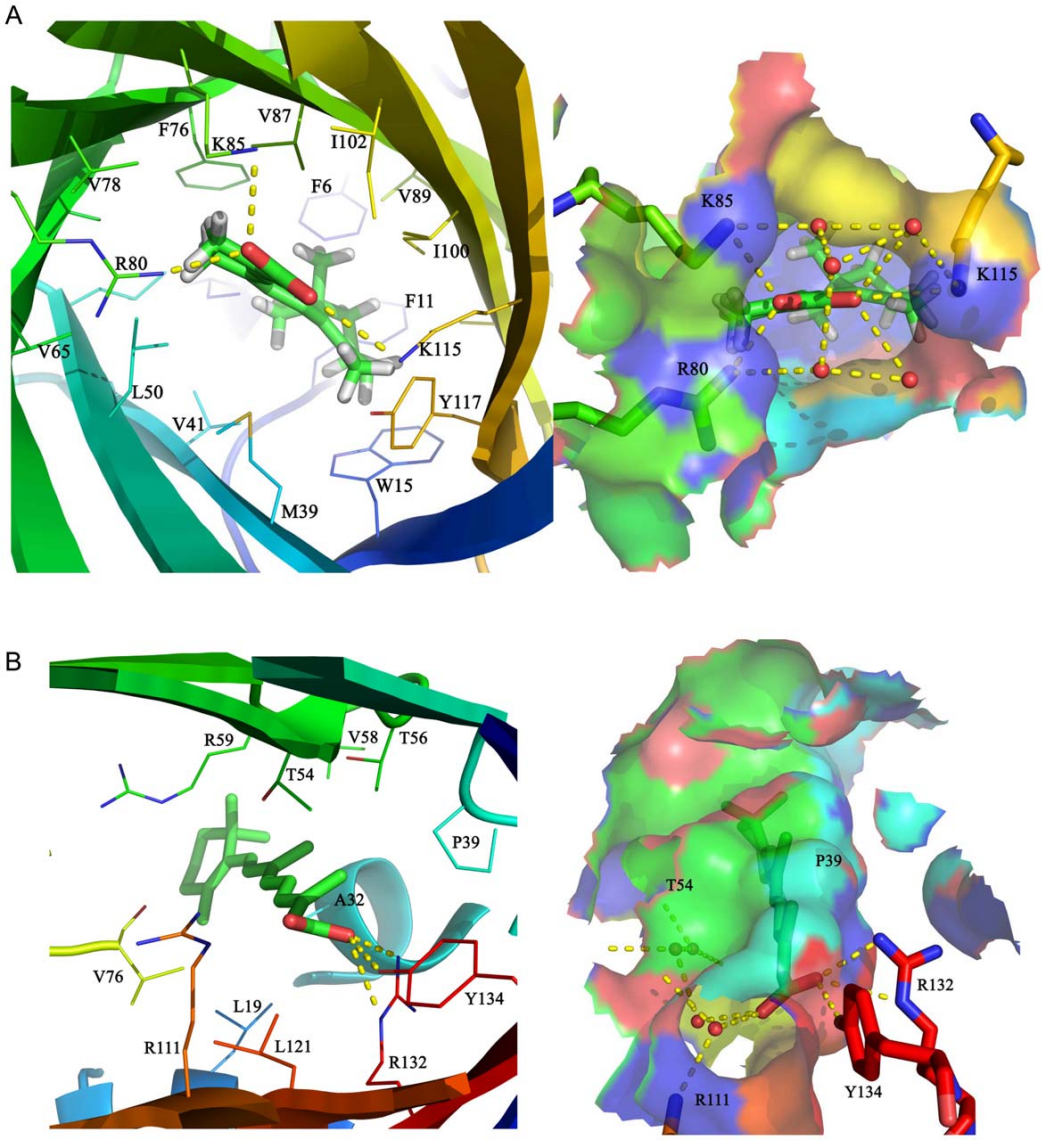
The retinoic acid-binding cavity in ERABP and CRABP. The retinoic acid binding sites are displayed and colored as in Figure 2. (A) In ERABP, three positively charged amino acids (Arg80, Lys85 and Lys115) along with the retinoic acid carboxylate form a network of three ion pairs at the entrance end of the amphipathic binding site. Additional polar side chains and water molecules that participate in the network are included. (B) The carboxylate of the ligand interacts with a trio of residues (Arg132, Tyr134 and Arg111) in CRABP.

The ligand binds deeply in the β-barrel of ERABP. The binding site in ERABP is complementary to the amphipathic ligand in both shape and chemical nature and largely excludes water in contrast to what is described for the retinoic acid binding site of the cellular retinoic acid binding protein [13]. There is a large amphipathic cavity inside the β-barrel, which forms the binding site and the binding site entrance (Fig. 3A). The charge network is located at the carboxylate end of the retinoic acid binding site. Three positively charged amino acids (Arg80, Lys85 and Lys115) and two negatively charged amino acids (Glu17 and Glu63) along with the retinoic acid carboxylate form a network of three paired ions at the entrance end of the amphipathic binding site. Additional polar side chains and water molecules also participate in the network. In the interior, these side chains make van der Waals contact with the ligand around the perimeter of the β-ionone ring. Sequence alignment shows that the amino acids involved in ligand binding are conserved in ERABP, except that Arg80 changes to Lys, which does not affect the protein-ligand polar interaction.

Two forms of CRABP have been identified with distinct tissue distributions and ligand specificities [2]. CRABP I appears to have a somewhat higher binding affinity for RA than CRABP II does. The amino acids in the binding sites of CRABP I and II are highly conserved among different species, thus we chose CRABP II as a case to analyze the ligand binding. CRABPs have extensive interactions with retinoic acid. These residues are shown in Figure 3B. The carboxylate of the ligand interacts with a trio of residues (Arg132, Tyr134 and Arg111). Water molecules also participate in the protein-ligand polar interaction network.

### Conclusion

Current knowledge regarding the metabolism of naturally occurring retinoids has been summarized in this paper. Dietary provitamin A carotenoids are largely converted to retinol (vitamin A) during intestinal absorption in mucosal cells. Newly absorbed vitamin A is stored in the liver as retinol and then displays two distinct functions through different conversion, which is essential for vision when it is converted to retinal (retinaldehyde), as well as participates in gene transcription when it is converted to retinoic acid [19]. Retinoic acid can be produced in the body by two sequential oxidation steps that convert retinol to retinaldehyde and further to retinoic acid, and this conversion is irreversible. All-trans retinoic acids are synthesized enzymatically from all trans-retinals and bind as ligand to the retinoic acid receptor family, which regulates genes transcription [20]. Retinoic acid acts by binding to the heterodimer of retinoic acid receptor (RAR) and the retinoid X receptor (RXR), which then bind to retinoic acid response elements (RAREs) in the regulatory regions of direct target genes (including Hox genes), thereby activating gene transcription [21].

Vitamin A is mobilized from liver stores and transported in plasma as retinol bound to a specific transport protein, called retinol-binding protein (RBP), which delivers retinol to peripheral target tissues. To specifically transport its ligand, RBP interacts with a number of intermoleculars, such as RBP carrier protein, TTR, to form a complex, which is proposed to be the vehicle for specific interaction with a putative cell surface receptor that mediates retinol uptake [22]. The existence of RBP receptor is supported by a large body of evidences. The hypothesis that has been advanced by Sundaram et al. [23] and other subsequent reviews involves a high-affinity retinol binding form of RBP that interacts with its receptor and releases retinol to the transport mechanism [24]. Given the potent biological effects (e.g., toxicity) of vitamin A and its derivatives, the controlled release of vitamin A into cells from holo-RBP through receptors [25] has an evolutionary advantage over the nonspecific diffusion of vitamin A. This mechanism makes it possible to achieve high efficiency and specificity for vitamin A delivery to organs distant from the liver, such as the eye, the brain, the placenta, and the testis [15], [26].

The RBP receptor on the cell surface not only specifically binds to RBP but also mediates vitamin A uptake from vitamin A-loaded RBP (holo-RBP). In doing so, RBP assumes a lower affinity form, which can readily be replaced on the receptor [15], [26]. To test this hypothesis, we performed molecular dynamics simulations to calculate the binding energy using the Discovery studio client 2.5 with the CDOCKER [27] protocol. We obtained the ten lowest energy conformations (Table S1). A significant rank sum test using MATLAB results shows that the energy of CRBP binding retinol (average of −81.24 kcal/mol) is lower than that of RBP (average of −86.83 kcal/mol, p = 1.83×10^−4^). This is in disagreement with Redondo and Vouropoulou's assumption. Our dynamics simulations also show that the average retinoic acid binding energies in ERABP and CRASP are −105.47 kcal/mol and −226.28 kcal/mol, respectively, which are higher than that of retinol (p = 1.83×10^−4^). This may account for the polar interactions between retinoic acid and transport proteins.

**Figure 4:**
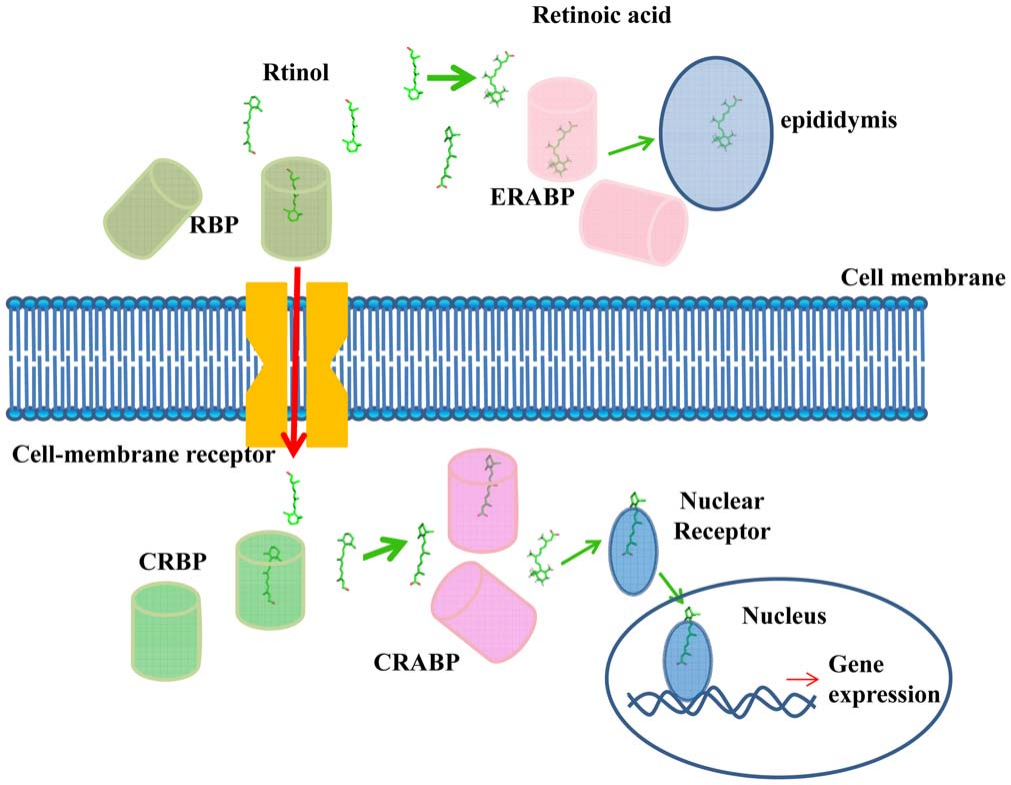
The retinoid possible transport model. In plasma, retinol binds with RBP in a higher-affinity form. Some of the retinol is oxidized to retinoic acid in the epididymis, which is required for sperm maturation. Most of the plasma retinol is transported to the interior of target cells across and across the cell membrane by a specific receptor. The retinol is picked up from the membrane by an intracellular structural homolog, called cellular retinol-binding protein (CRBP), in a lower-affinity form. Once inside the cells, the low affinity form may be readily used by the cell. In different subcellular locations, the retinol binding orientation is reversed. When the body is in need of vitamin A, the retinol dissociates from the CRBP, which is converted into retinoic acid and bound by CRABP. CRABP then transports retinoic acid to the nucleus across the nuclear receptor, thereby activating gene transcription.

Considering these findings, we propose a possible model for retinoid transport throughout the body (Fig. 4). In plasma, retinol binds with RBP in a higher-affinity form. Some retinol is oxidized to retinoic acid in the epididymis, which is required for sperm maturation. Most of the retinol in plasma is transported to the interior of target cells through acrossing the cell membrane via a specific receptor. And then in cell, retinol is picked up by an intracellular structural homolog, called cellular retinol-binding protein (CRBP), in a lower-affinity form, which may facilitate its use by the target cell. In different subcellular locations, the retinol binding orientation is reversed. When the body is in need of vitamin A, retinol dissociates from CRBPs and is converted into retinoic acid and then bound by CRABP. CRABP then transports retinoic acid to the nuclear receptors, thereby activating gene transcription. This process involves many protein-protein interactions, and conformational changes may be an integral part of the retinoid transfer mechanism. Moreover, the dissociation of retinol from CRBP appears to require the assistance of an external factor. The targeted release of retinol *in vivo* is likely to be promoted by the properties of the microenvironment near the membranes where the enzyme molecules that are involved in its metabolism are embedded. In conclusion, although the crystal and solution structures, as well as the backbone dynamics of various intracellular retinoid carriers in the apo- and holo-form provide fundamental information, they do not lead to a common mechanism of ligand exchange. In particular, the different levels of accessibility to the cavity might be the result of fine tuning of the protein conformation, which is made possible by the limited differences between the sequences and is likely to be required to optimize each carrier protein's physiological function [10].

In the process of evolution, similar protein architectures can be adapted to bind similar ligands in completely different ways [14]. Retinoid transport proteins are a good example of this principle. In the same superfamily, RBP, ERABP, CRBPs and CRABPs may descend from a common ancestor. Additionally, in different locations within the cell their structure and binding mechanisms have changed during evolution.

## Materials and Methods

### Date collection

We chose the PDB codes for 1BRP, 1EPB, 1CRB and 1CBS as examples of the RBP, ERABP, CRBPs and CRABPs families of proteins, respectively. The structures and sequences were retrieved from the Protein Data Bank (PDB) [28]. We then used the four stand-alone PSI-BLAST sequences [29] to search against the non-redundant (NR) database (iteration = 5, b = 1000, others are default) and retrieve the sequences. The binding site amino acids were collected using the notation of the PDBsum database [30], [31].

### Sequence, structure alignment and binding amino acid conservation analyses

The sequences of each transport protein were aligned by CLUSTALW [32]. Pairwise protein structure alignments were performed with the CE [33] program. The structure and binding site analyses were performed with the Discovery studio client 2.5 program using standard parameters.

### Molecular dynamics simulation and statistical analysis

Molecular dynamics (MD) simulations were performed using CDOCKER [27], a CHARMm-based MD docking algorithm in Discovery Studio (v2.5.0.9164), and the top 10 conformations for every protein were used to calculate the binding energy.

To perform these docking studies, the four retinoid protein-ligand complexes were collected from the PDB database (IDs are 1BRP, 1EPB, 1CRB and 1CBS). The protein is kept rigid while the ligands are treated as fully flexible. Random conformations of the ligands are generated using high-temperature MD. The conformations are then translated into the binding site. Candidate conformations are then created using random rigid-body rotations followed by simulated annealing. A final minimization step is applied to each of the ligand's docking conformations by using a CHARMm-based molecular dynamics (MD) scheme for 100 picoseconds. In this process, the heating target temperature was set to 700 K, and the cooling target temperature was set to 300 K. These minimized docking conformations were then clustered based on a heavy atom RMSD approach using a 1.5 Å tolerance. The ranking of the ligand's docking conformations was based on the total docking energy (including the intermolecular energy for ligands and the ligand-protein interactions). For every protein, the top ten conformations were used to calculate the receptor-ligand binding free energies.

A statistical significance analysis between the pairs of each protein-ligand binding energy data was performed using a rank-sum test in Matlab (version 7.13.0.564).

## Supporting Information

Figure S1 **Multiple sequence alignment of four types CRBPs.** Conserved amino acid residues are boxed in different color by amino acid attribute. In four types CRBPs, the retinol binding site residues are either identical or chemically conserved in CRBP I, II and CRBP III and CRBP IV. The only exception is Q108 (the star noted in picture), a residue whose amide group hydrogen bonds the alcoholic group of retinol in CRBP I and most CRBP II proteins. In chicken and xenopus laevis, CRBP II at amino acid 108 was mutated to histidine. The Q108 residue is replaced by histidine in CRBP III and CRBP IV.
(PDF)

Table S1 **The binding energy between retinoids binding proteins and their ligands.**
(PDF)

Table S2 **The sequence identity of four retinoids binding proteins.**
(PDF)

Table S3 **The value of RMSD of CE structure alignment.**
(PDF)
