# Supplementary material for: Retinoid-Binding Proteins: Similar Protein Architectures Bind Similar Ligands via Completely Different Ways
Source: PLoS One. 2012 May 4;7(5):e36772. doi: 10.1371/journal.pone.0036772 (PMC3344936; doi:10.1371/journal.pone.0036772)
Supplement: Table S1 — The binding energy between retinoids binding proteins and their ligands. (PDF) [file pone.0036772.s002.pdf]

Table S1. The binding energy between retinoids binding proteins and their ligands

Protein	RBP(1brp)	CRBP(1crb)	ERABP(1epb)	CRABP(1cbs)
Top ten Binding Energy (kcal/mol)	-87.21842	-81.88952	-109.37775	-229.07327
	-87.21996	-81.88583	-106.39604	-229.07442
	-87.22162	-81.10282	-118.44061	-233.66422
	-87.22178	-81.10774	-105.52261	-233.81225
	-87.22025	-81.10824	-94.60466	-229.19979
	-87.22163	-81.11019	-108.46828	-221.4997
	-87.22008	-81.10822	-105.38868	-221.49902
	-82.28446	-80.90537	-106.39851	-221.49894
	-87.97112	-81.10728	-105.52446	-221.49862
	-87.53759	-81.10772	-94.60132	-222.01486
Average	-86.8337	-81.2433	-105.472	-226.284
