# Supplementary material for: Retinoid-Binding Proteins: Similar Protein Architectures Bind Similar Ligands via Completely Different Ways
Source: PLoS One. 2012 May 4;7(5):e36772. doi: 10.1371/journal.pone.0036772 (PMC3344936; doi:10.1371/journal.pone.0036772)
Supplement: Table S3 — The value of RMSD of CE structure alignment. (PDF) [file pone.0036772.s004.pdf]

Table S3. The value of RMSD of CE structure alignment

Protein name	RBP	ERABP	CRBP	CRABP
PDB ID	1brp	1epb	1crb	1cbs
Amino acid number	182AA	164 AA	134 AA	137 AA
RBP	-			
ERABP	2.3	-		
CRBP	4.4	3.2	-	
CRABP	4.2	3.2	1.5	-
