# Supplementary material for: Retinoid-Binding Proteins: Similar Protein Architectures Bind Similar Ligands via Completely Different Ways
Source: PLoS One. 2012 May 4;7(5):e36772. doi: 10.1371/journal.pone.0036772 (PMC3344936; doi:10.1371/journal.pone.0036772)
Supplement: Figure S1 — Multiple sequence alignment of four types CRBPs. Conserved amino acid residues are boxed in different color by amino acid attribute. In four types CRBPs, the retinol binding site residues are either identical or chemically conserved in CRBP I, II and CRBP III and CRBP IV. The only exception is Q108 (the star noted in picture), a residue whose amide group hydrogen bonds the alcoholic group of retinol in CRBP I and most CRBP II proteins. In chicken and xenopus laevis, CRBP II at amino acid 108 was mutated to histidine. The Q108 residue is replaced by histidine in CRBP III and CRBP IV. (PDF) [file pone.0036772.s001.pdf]

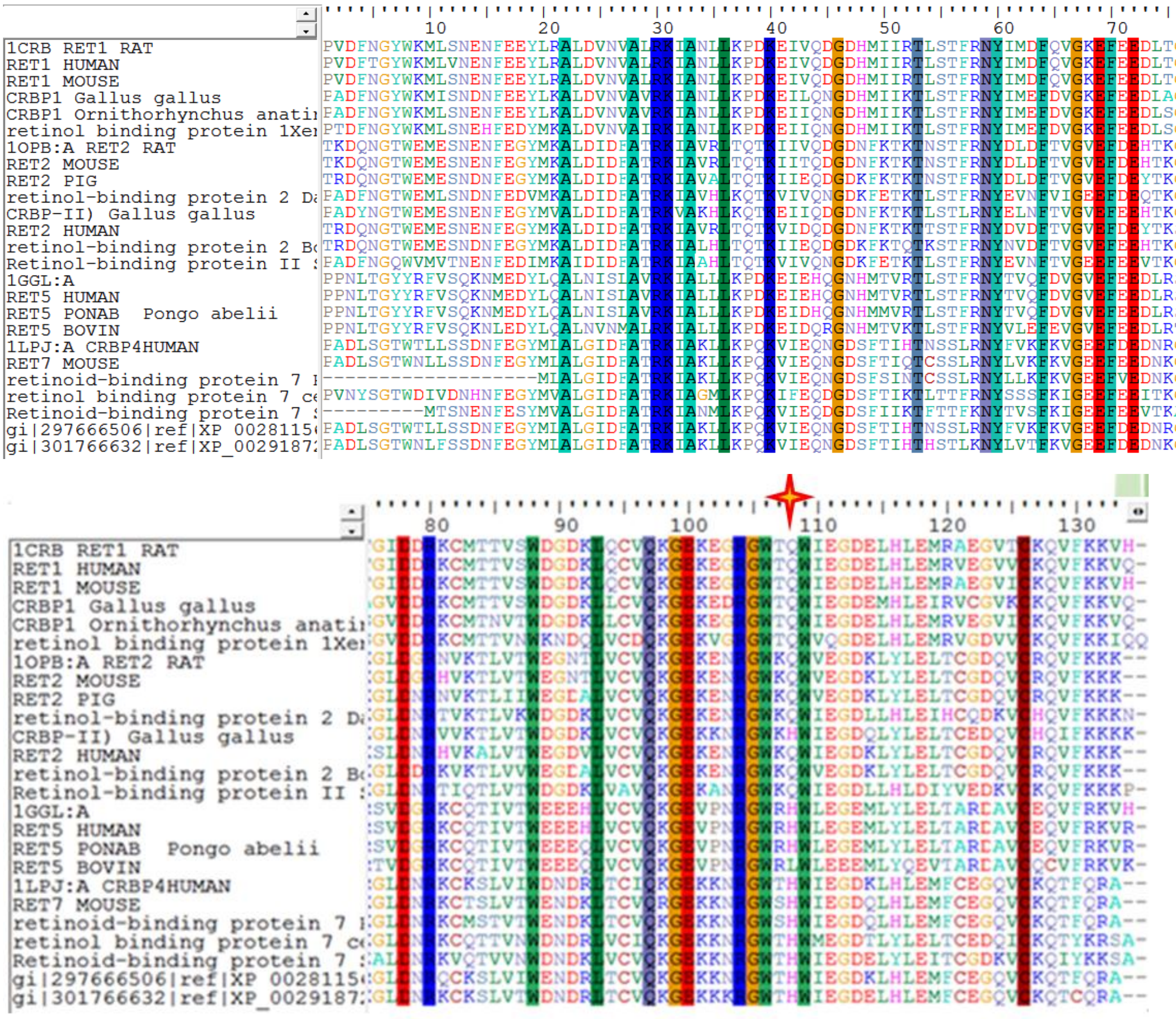

Figure S1. Multiple sequence alignment of four types CRBPs. Conserved amino acid residues are boxed in different color by amino acid attribute. In four types CRBPs, the retinol binding site residues are either identical or chemically conserved in CRBP I, II and CRBP III and CRBP IV. The only exception is Q108 (the star noted in picture), a residue whose amide group hydrogen bonds the alcoholic group of retinol in CRBP I and most CRBP II proteins. In chicken and xenopus laevis, CRBP II at amino acid 108 was mutated to histidine. The Q108 residue is replaced by histidine in CRBP III and CRBP IV.
